# Immunity Against *Leishmania major* Infection: Parasite-Specific Granzyme B Induction as a Correlate of Protection

**DOI:** 10.3389/fcimb.2018.00397

**Published:** 2018-11-13

**Authors:** Thouraya Boussoffara, Sadok Chelif, Melika Ben Ahmed, Mourad Mokni, Afif Ben Salah, Koussay Dellagi, Hechmi Louzir

**Affiliations:** ^1^Laboratory of Transmission, Control and Immunobiology of Infections, Pasteur Institute of Tunis, Tunis, Tunisia; ^2^Université de Tunis El Manar, Tunis, Tunisia; ^3^Department of Dermatology, Hospital La Rabta, Tunis, Tunisia

**Keywords:** *Leishmania major*, specific-cytotoxicity, granzyme B, correlate of protection, re-infection

## Abstract

Zoonotic cutaneous leishmaniasis (ZCL) caused by *Leishmania (L.) major* infection is characterized by different clinical presentations which depend in part on the host factors. In attempt to investigate the impact of the host's immune response in the outcome of the disease, we conducted a prospective study of 453 individuals living in endemic foci of *L. major* transmission in Central Tunisia. Several factors were assessed at the baseline including (i) the presence of typical scars of ZCL, (ii) *in vivo* hypersensitivity reaction to leishmanin, and (iii) the *in vitro* release of granzyme B (Grz B) by peripheral blood mononuclear cells (PBMC) in response to stimulation with live *L. major* promastigotes. After one season of parasite's transmission, repeated clinical examinations allowed us to diagnose the new emerging ZCL cases. Heterogeneity was observed in terms of number of lesions developed by each individual as well as their size and spontaneous outcome, which led us to establish the parameter “severity of the disease.” The efficacy of the presence of typical ZCL scar, the leishmanin skin test (LST) positive reactivity and the high levels of Grz B (≥2 ng/ml), in the protection against the development of ZCL were 29, 15, and 22%, respectively. However, these factors were more efficient against development of intermediate or severe forms of ZCL. Levels of Grz B >2 ng/ml showed the best efficacy of protection (equals to 72.8%) against development of these forms of ZCL. The association of such parameter with the positivity of the LST exhibited a better efficacy (equals to 83.6%). In conclusion, our results support the involvement of *Leishmania*-specific cytotoxic cellular immune response in host protection against *Leishmania*-infection. This factor could be of great interest in monitoring the success of vaccination against human leishmaniasis.

## Introduction

Cutaneous leishmaniasis are parasitic infections caused by different species of *Leishmania* parasite and grouping distinct clinical manifestations. In Tunisia, ZCL is due to infection by the parasite *L*. *major* zymodeme MON-25 and transmitted by *Phlebotomus papatasi* (Ben Ismail and Ben Rachid, 1989). ZCL takes place as seasonal epidemics with an annual prevalence ranging from 2 to 10 thousand cases (Bettaieb et al., 2014; Bettaieb and Nouira, 2017). Transmission of the parasite occurs during the summer months, and the emergence of active lesions in humans is recorded during the autumn and winter months. The clinical features of ZCL are rather polymorphic, ranging from benign self-limited to wide cutaneous lesions that may cause severe disfigurement. Nevertheless, human's infection by *Leishmania* might be asymptomatic, particularly in endemic areas (Ben Salah et al., 2005). The outcome of the *Leishmania*-infection depends partly on the type and intensity of the host immune response. Moreover, the *Leishmania*-specific immunity associated with healing often provides a resistance to subsequent infection (Guirges, 1971; Davies et al., 1995).

Healing of cutaneous leishmaniasis was mostly associated with the development of a type 1 CD4^+^ cell-specific immune response (Sassi et al., 1999) as well as a positive leishmanin skin test (LST) reactivity (Liew and O'Donnell, 1993; Reithinger et al., 2007). Therefore, vaccine efficacy is currently evaluated through *Leishmania*-specific Delayed Type Hypersensitivity (DTH), PBMC proliferation, or interferon (IFN)-γ production in response to stimulation with *Leishmania* antigens. These indicators of Th1 response were usually used for the selection of naïve individuals and as correlate of protection during the clinical trials (Reithinger et al., 2007; Duthie et al., 2012). LST is usually used for clinical diagnosis of leishmaniasis and epidemiological surveys (Ben Salah et al., 2005). It is considered as a good protective correlate when evaluating the efficacy of vaccines against leishmaniasis in humans (de Luca et al., 2001; Alimohammadian et al., 2002). However, previous trials evaluating anti-*Leishmania* vaccines, consisting of the heat-killed or autoclaved *L. major* parasite showed that there is no protective effect despite the conversion of the LST in vaccinated individuals (Sharifi et al., 1998; Khalil et al., 2000; Armijos et al., 2004). Obviously, the positive LST reactivity observed after vaccination with killed parasite is generally not predictive of protection against ZCL, despite a lower prevalence of disease in individuals with positive LST. Thus, although LST conversion might be an indicator of *Leishmania*-specific immunity, it may not, however, be considered as an accurate correlate of protection against the disease (Momeni Boroujeni et al., 2013), hence the need to define the immunological basis of resistance to infection with *Leishmania* parasites. Besides the key role of CD4^+^ Th1 cells, which is closely associated with LST reactivity, several studies pointed to the involvement of CD8^+^ T cells in acquiring immunity against leishmaniasis in mice model (Belkaid et al., 2002; Rhee et al., 2002). Such cells mediate effectors mechanisms through the secretion of cytokines and chemokines and also through cytotoxic activity (Ruiz and Becker, 2007). Several studies have shown that human *Leishmania*-specific cell-mediated cytotoxicity is part of the *Leishmania*-specific acquired immunity (da Conceição-Silva et al., 1994; Brodskyn et al., 1997; Marry et al., 1999; Russo et al., 1999; Bousoffara et al., 2004). Accordingly, in a previous study, we have demonstrated a cytotoxicity of peripheral blood lymphocytes toward *L. major*-infected macrophages as well as a significant increase of Grz B activity in patients with active infection or those healed from ZCL (Bousoffara et al., 2004). The role of cytotoxic cells during cutaneous leishmaniasis is still controversial. Indeed, it seems that cytotoxic activity might contribute to infection clearance but also to skin ulcer development in patients with CL (Stäger and Rafati, 2012).

In attempt to evaluate the impact of host *Leishmania*-specific cytotoxic immune response on the outcome of ZCL, we have conducted a prospective study of a large cohort of subjects living in endemic foci in Tunisia. Elucidation of the specific effective immunological mechanisms responsible for the resistance to leishmaniasis is crucial for vaccine development and evaluation.

## Materials and methods

### Study population and samples

A total of 59 subjects have been enrolled in the first part of this study consisting on the optimization of Grz B test. Subjects were categorized on four groups basing on clinical, epidemiological and immunological criteria:

- 15 patients with active ZCL (number of ZCL lesion ranging from 1 to 7) living in the endemic region of Kairouan. All of these patients showed a positive LST reactivity.
- 24 subjects healed from ZCL living in the endemic regions of Sidi Bouzid or Rmada, characterized by the presence of typical ZCL scars and a positive LST reactivity and/or a positive lymphoproliferative response to soluble *Leishmania* antigens (SLA).
- 11 healthy subjects living in the endemic focus of Rmada selected basing on the absence of ZCL scars and the negative LST reactivity.
- 9 healthy subjects (Volunteers working in the laboratory of transmission, control, and immunobiology of infections at Pasteur Institute of Tunis) living outside endemic area of *L. major* transmission. These individuals were enrolled in the study basing on the absence of ZCL scars and the negativity of *Leishmania*-specific lymphoproliferative response (Δcpm< 5,000). Table 1 shows detailed description of this population.

**Table 1:** Clinical and immunological data of subjects included in the study.

	**ZCL patients (*n = 15*)**	**Healed ZCL subjects (*n = 24*)**	**Healthy subjects living in endemic area (*n = 11*)**	**Healthy subjects living outside endemic area (*n = 9*)**
Age, mean ± SD (min-max), year	11.5 ± 2.5 (7–16)	13.7 ± 1.16 (12–16)	13.45 ± 1.57 (11–16)	24.78 ± 4.29 (20–30)
Male/Female	6/9	10/14	6/5	6/3
Number of lesion, mean (min-max)	2,6 (1–7)	0	0	0
ZCL scar^a^	0/15	24/24	0	0
LST^b^ Induration's diameter, mean ± SD (min-max), mm	10/11 7.6 ± 3.1 (0–15)	22/22 10.22 ± 2.5 (5.5–15.5)	0/11 0	ND
*Leishmania*-specific lymphoproliferative response^c^	8/9	20/23	1/9	0/9

*LST, Leishmanin Skin Test; ZCL, Zoonotic Cutaneous Leishmaniasis; ND, Not done*.

a *Number of subject with ZCL scar/Total number of subject tested*.

b *Number of subject with positive LST/Total number of subject tested*.

*Positive LST, Induration's diameter ≥5 mm*.

c *Number of subject with positive Leishmania-specific lymphoproliferative response (Δcpm ≥ 5,000)/Total number of subject tested*.

**Figure 1:**
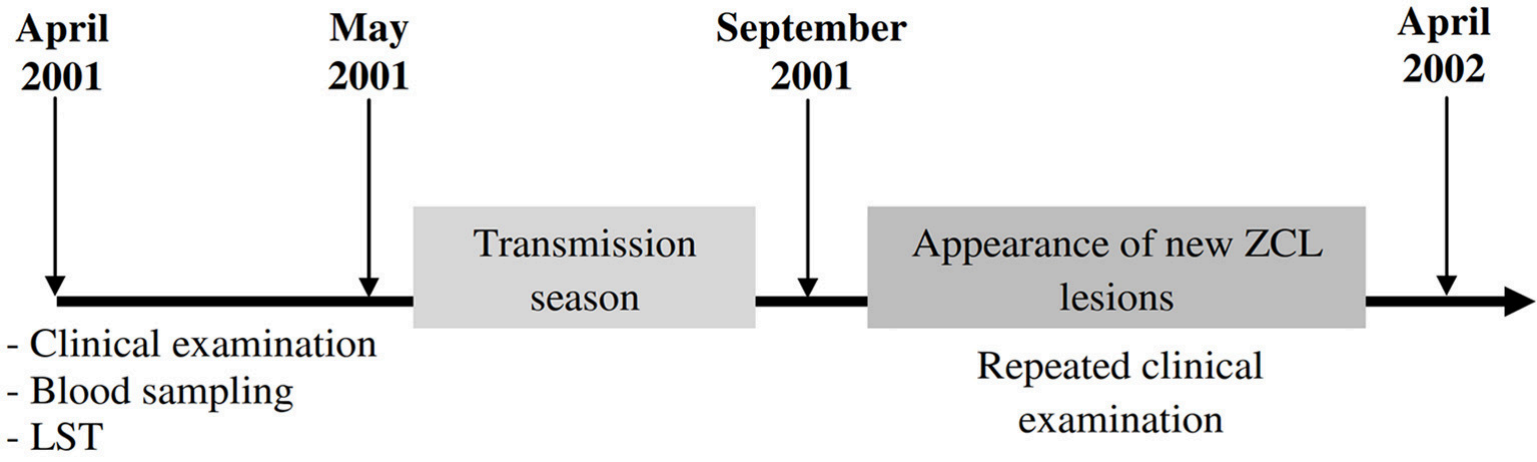
Timeline showing progress of the prospective study. Four hundred and fifty three participants living in endemic foci of zoonotic cutaneous leishmaniasis (ZCL) in Central and Southwestern Tunisia (Gafsa and Sidi Bouzid) were followed up throughout one season of *L. major* transmission. Several parameters such as leishmanin skin test (LST) or the presence of typical ZCL scar were monitored at the beginning of the study. Peripheral blood samples were obtained from each donor and will be used to analyze host cytotoxic immune response. Repeated clinical examinations were done to diagnose the new cases of ZCL emerging after one transmission season (September 2001–April 2002).

The second part consists on a prospective study carried out in two different endemic foci for *L. major* (Zymodeme MON-25) infection in Tunisia: an old endemic focus (OEF) situated in the district of El Gtar, governorate of Gafsa (south west Tunisia); and a new endemic focus (NEF) situated in the district of Souk Ejjdid, governorate of Sidi Bouzid (Central Tunisia). This study was carried out as part of the research project “Advances in epidemic parameters of ZCL to validate tools for surveillance and control.” At enrolment of the study population between April and May 2001, before the parasite transmission season, the donors had a clinical examination looking for active ZCL lesions or typical scars, a blood sampling and an intradermal administration of leishmanin for LST reactivity. Repeated clinical examinations were performed during the season of emergence of lesions (between September 2001 and April 2002) to evaluate the clinical course of *L. major* infection, occurring after the transmission season (Figure 1). The protocols were approved by the institutional review board as detailed below. A total of 453 subjects were enrolled in this study (age, mean ± SD: 7.82 ± 1.9 years [range, 3–15 years]; male: female ratio, 0.96). The study population was selected from four primary schools in the district of El Gtar (*n* = 212) and from three primary schools in the district of Souk Ejjdid (*n* = 187). Children <6 years old and therefore, of pre-school age, from the region of souk Ejjdid were also recruited (*n* = 54; Table 2).

**Table 2:** Clinical and immunological data of subject included on the prospective study.

	**Subject living within OEF (*n* = 212)**	**Subject living within NEF (*****n*** = **241)**		**Total (*n* = 453)**
		**Age ≥ 6 years (*n* = 187)**	**Age < 6 years (*n* = 54)**	
Age, mean ± SD(min-max), year	7.9 ± 1.5 (6–15)	8.68 ± 1.42 (6–12)	4.4 ± 0.66 (3–5)	7.82 ± 1.9 (3–15)
Sex ratio (Male/Female)	0.96 (104/108)	0.85 (85/100)	1.57 (33/21)	0.96 (222/229)
ZCL scar^a^	60/212	36/186	9/54	105/452
LST^b^	72/212	80/186	15/54	167/452
Diameter of induration, mean ± SD	3.6 ± 5.2	4.5 ± 4.5	3.45 ± 3.79	4 ± 4.82
(min-max), mm	(0–31)	(0–15)	(0–13)	(0–31)

LST, Leishmanin Skin Test;

a *Number of subject with scars/Total number of subject tested*.

b *Number of subjects with positive LST/Total number of subject tested*.

*OEF, Old endemic foci; NEF, New endemic foci*.

### Ethics statements

Before starting the study, written consent were obtained from participants or from their parents in case of children. Subjects enrolled in this study were verbally informed of the nature of the research project and written consent was obtained for the clinical follow-up, leishmanin skin test, and blood sampling. Consent was prepared in the native language (Arabic). The protocol was approved by the Bio-Medical Ethics Committee (BMEC) of Pasteur Institute of Tunis. The leishmanin test applied to the study population is made of leishmanin approved by WHO norms and regulation. It was previously used for thousands of individuals worldwide without significant hazards.

### Leishmanin skin test

LST was performed by intradermal injection of 100 μL of leishmanin suspension as previously described by Sassi et al. (1999). An induration's diameter ≥5 mm indicates a positive LST reactivity.

### Parasite culture

In the present study, we used *L. major* (zymodeme MON25; MHOM/TN/94/GLC94) obtained from a human ZCL lesion (Kébaïer et al., 2001). Parasites were cultivated at 26°C in RPMI 1640 medium (Sigma, St. Louis, MO) containing 2 mM L-glutamine, 100 U/mL penicillin, 100 mg/mL streptomycin, and 10% heat-inactivated fetal calf serum. Stationary-phase promastigotes were used for preparation of soluble *Leishmania* antigens (SLA) as previously described (Sassi et al., 1999) and for stimulation of PBMCs.

### Lymphoproliferative test

PBMCs were separated from heparinized blood samples using Ficoll-Paque (GE Healthcare) density gradient centrifugation. PBMCs were cultured in 96-well plates at a concentration of 1 × 10^6^ cells/mL in a final volume of 200 μL of RPMI 1640 medium (Sigma) supplemented with 2 mM L-glutamine (Sigma), 100 U/mL penicillin (Sigma), 100 μg/mL streptomycin (Sigma), and 10% heat-inactivated human AB serum (Sigma). PBMCs were stimulated with SLA (10 μg/mL) for 5 days and lymphoproliferative response was evaluated after adding 1μCi/well of [^3^H]-thymidine (Amersham, France) for the last 6 h using a liquid scintillation counter (Rack Beta, LKB Wallace, Australia). Results were expressed as Δcpm, difference between mean counts of triplicates in SLA-stimulated PBMC and mean counts of triplicates in unstimulated PBMC.

### Measure of granzyme B and IFN-γ level in culture supernatant

Measurement of Grz B levels was performed using ELISA described by Spaeny-Dekking et al. (1998). The Grz B captured by an anti-Grz B monoclonal antibody (MoAb GB11), fixed on microtiter plate was revealed by a biotin-labeled anti-Grz B monoclonal antibody (GB10 MoAb). Recombinant Grz B of known concentration was used for tracing a standard curve. Briefly, wells of the microtiter plates (Maxisorp, NUC) were coated overnight at 4°C with 100 μl/well of MoAb GB11 diluted on carbonate buffer, 0.1 M NaHCO_3_ pH 9.6, at 2 μg/ml. Plates were subsequently washed five times with PBS containing 0.02% Tween 20. The free sites were saturated by incubation for 45 min at room temperature (RT) with 150 μl/well of PBS containing 2% tween 20. The recombinant Grz B and the samples, diluted in HPE (high performance ELISA buffer, Central laboratory of the Red Cross Blood Transfusion Service) and 100 μl were added and incubated for 1 h at RT. After washing, 100 μL of GB10 MoAb diluted at a concentration of 0.5 μg/mL in HPE buffer containing 1% (v/v) of normal murine serum (NMS). After incubation for 1 h, the wells were washed and then incubated for 20 min with 100 μl of streptavidin coupled to peroxidase (Amersham, Saclay, France) diluted at 1/10,000. Five washes were carried out before adding 100 μl/well of TMB solution (3,3′, 5,5′ tetramethylbenzidine, Merck, Darmstadt, Germany) at a concentration of 100 μg/mL containing 0.003% (v/v) H_2_O_2_, diluted in 0.11M sodium acetate buffer pH 5.5. After stopping the reaction with 2N H _2_SO_4_, optical density was measured using an automated plate reader (Titerteck Multiscan) at 450 nm/540 nm. Grz B levels were determined by reference to the standard curve and results were expressed in pg/mL. Detection threshold was fixed at 1.42 pg/mL.

Measurement of IFN-γ level within supernatant was carried out using OptEIA set ELISA (BD Biosciences, San Jose, CA) according to the manufacturer's recommendations. The results were interpolated from the standard curve established using recombinant IFN-γ and expressed in pg/mL. Detection threshold was fixed at 6.02 pg/mL.

### Statistical analysis

Statistical analysis was performed using SPSS (version 20.0; SPSS) software. The association between two parameters was assessed by the Spearman's Rank Correlation Coefficient test. The correlation is considered statistically significant if *r* > 0.3 with a significance level *p* < 0.05.

The optimal and recommended cut-off points (where applicable) were used to calculate the numbers of subject positive and negative for each test. The comparison between two groups for a studied parameter was carried out by Student's *t*-test (for the cohort study) or by the Mann-Witney *U*-test (in the validation step).

For the prospective study, further analysis was performed, including the χ2 tests applied to analyze the associations between the categorical results (LST reactivity/Scar; LST/Grz B; Scar/Grz B). Odds ratios (OR) were then computed to determine the nature of the association between different parameters by stratifying subjects as described by van Belle et al. (2004). A Fisher's exact *P*-value was also obtained for each association. A positive association is indicated by an odds ratio >1 (significant when *p* < 0.05 and the 95% confidence limits both exceed 1). Odds ratios significantly <1 indicate a negative association. Estimation of the efficacy of the immune status of the host on the protection against the development of the ZCL was performed through the calculation of the relative risk (*RR*) and then the preventive fraction (Rothman et al., 2008). In our case, the analyzed factors consist of positive LST reactivity, presence of typical ZCL scar and high levels of Grz B (>2 ng/ml).

## Results

### Levels of Grz B produced by PBMCs stimulated with *L. major* promastigotes

**Figure 2:**
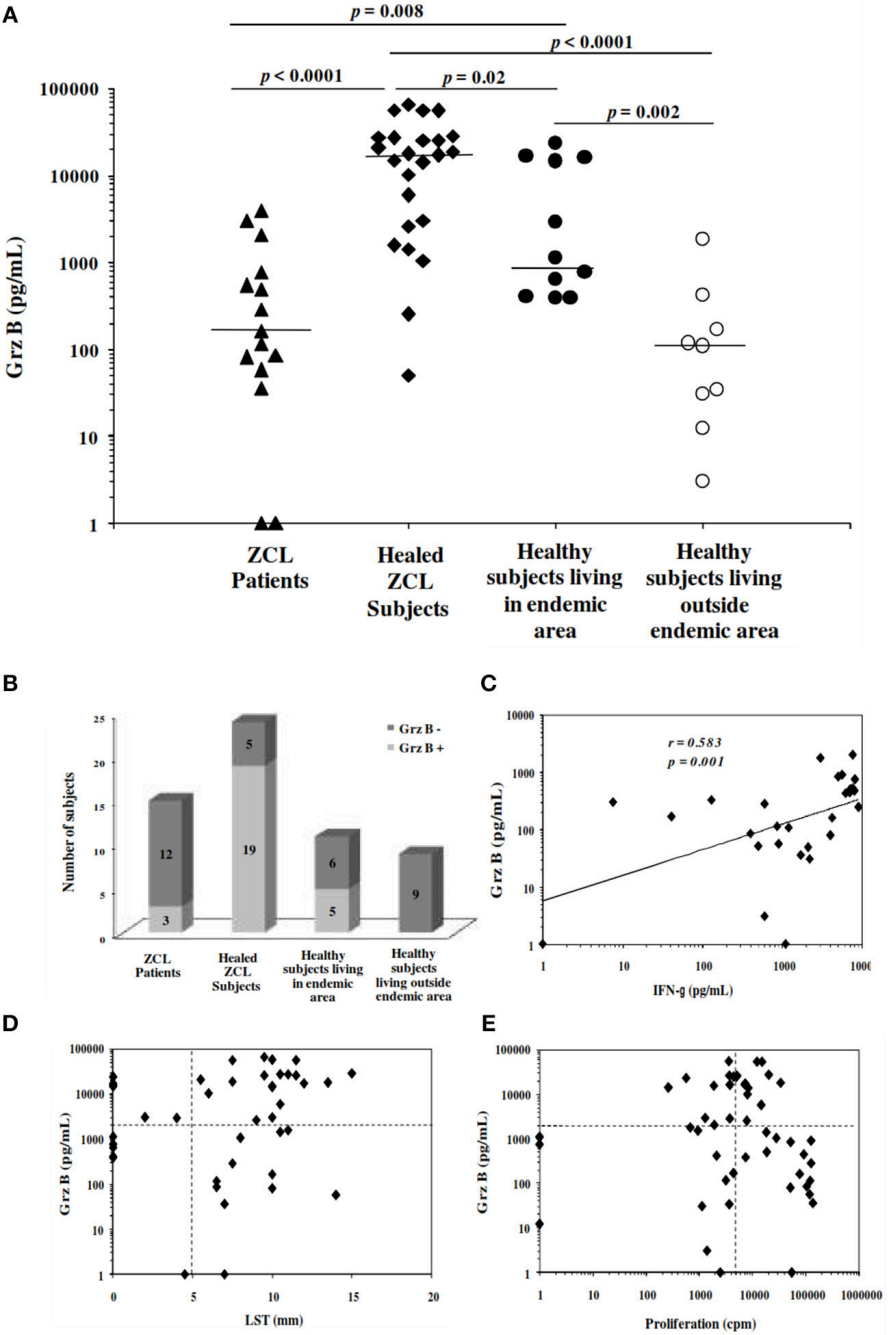
Granzyme B levels induced in response to stimulation with *Leishmania major***. (A)** Grz B is measured in culture supernatants of PBMCs incubated for 5 days with live promastigotes *L. major* at a ratio of 3 promastigotes per cell. We used PBMCs from patients with active ZCL, healed ZCL subjects or from healthy individuals living in endemic focus for transmission of *L. major* or outside these areas. Horizontal bars represent median values. **(B)** Histograms represent the number of subjects categorized in Grz B^+^ and Grz B^−^, basing on the cut-off fixed at 2,000 pg/mL. **(C)** Association between Grz B and IFN-γ levels measured in the same supernatants **(D,E)** Association between Grz B levels and results of leishmanin skin test (LST), expressed as diameter of induration and the lymphoproliferative response to soluble *Leishmania* antigens (SLA), expressed as Δcpm. Horizontal dashed lines indicate cut-off for Grz B level, LST and proliferation fixed at 2,000 pg/ml, 5 mm and 5,000 cpm, respectively.

Before starting the prospective study, we optimized the conditions for the analysis of *Leishmania*-specific cytotoxic immune response. This was done by measurement of Grz B level in supernatants of PBMCs stimulated for 5 days with promastigotes of *L. major* at a ratio of 3 parasites per cell. The analysis was carried out for 15 subjects with active ZCL, 24 healed ZCL subjects selected basing on the presence of typical scar and positive reactivity to LST (Scar^+^LST^+^), and a total of 20 apparently healthy donors. The latter group includes 11 subjects living in endemic areas with no history of ZCL and negative LST (Scar^−^LST^−^), and 9 subjects living outside these area (Table 1). As shown in Figure 2A, the levels of Grz B detected within supernatants of PBMC from subjects cured of the disease (mean ± SD: 20693.9 ± 19576.8 pg/mL) were significantly higher compared to those measured in ZCL patients (mean ± SD: 779.62 ± 1231.48 pg/mL; *p* < 0.001). In addition, levels of Grz B measured in culture supernatants of cells from apparently healthy and naïve (Scar^−^LST^−^) individuals but living in endemic region of *L. major* transmission were significantly higher (mean ± SD: 7088 ± 8837.11 pg/mL) compared to those detected in the supernatants of PBMCs from healthy and naïve individuals living outside endemic areas (mean ± SD: 305.06 ± 589.67 pg/mL; *p* = 0.002). This result is very important and may suggest that in endemic area, even in the absence of LST reactivity other marker of specific immune response (like Grz B production) could indicate a previous contact with the *Leishmania* parasite.

We thus used the results obtained with healthy and naïve individuals living outside endemic areas as reference for the definition of the cut-off of positivity for Grz B levels and calculated as the mean + 3 times the standard deviation of the Grz B levels produced by PBMCs from healthy negative controls living outside endemic area. According to the cut-off, fixed at 2,000 pg/ml, we found that three among the 15 subjects with active ZCL, 19 among the 24 subjects with a history of ZCL and five among the 11 healthy individuals (Scar^−^LST^−^) living in endemic area were Grz B^+^. However, none of the healthy negative controls living outside endemic areas had Grz B levels greater than the cut-off (Figure 2B).

Otherwise, a positive correlation was found between Grz B levels and those of IFN-γ measured in culture supernatants (Spearman rank correlation coefficient *r* = 0.583, *p* = 0.001; Figure 2C). Furthermore, a concordance of 61.36 and 45.09% was found between Grz B levels and results of LST and SLA-specific lymphoproliferation, respectively (Figures 2D,2E).

### Evaluation of the Grz B production as marker of cytotoxic immune response for subjects included in a prospective survey

In a next step, we evaluated the *Leishmania*-specific cytotoxic immune response at the baseline (before the transmission season of the parasite) within donors included in the prospective study (*n* = 453). Results of the clinical examination as well as those of the LST collected at the baseline showed that 167 subjects had a positive LST, among them 75 individuals exhibited ZCL scars (Table 2). Among the remaining individuals with a negative LST, 30 individuals showed ZCL scars. Interestingly, the percentage of individuals with ZCL scars is higher in the old focus (28%; 60/212) compared to the new one (18.7%; 45/240). In addition, similar percentages of subjects with positive LST reactivity were observed in both of focus (33.9% in OEF and 39.5% in NEF). However, in the NEF, this percentage is lower within subjects under 6 years old (27%) compared to that of subjects older than 6 years (43%) indicating a limited contact with the parasite for this age group.

**Figure 3:**
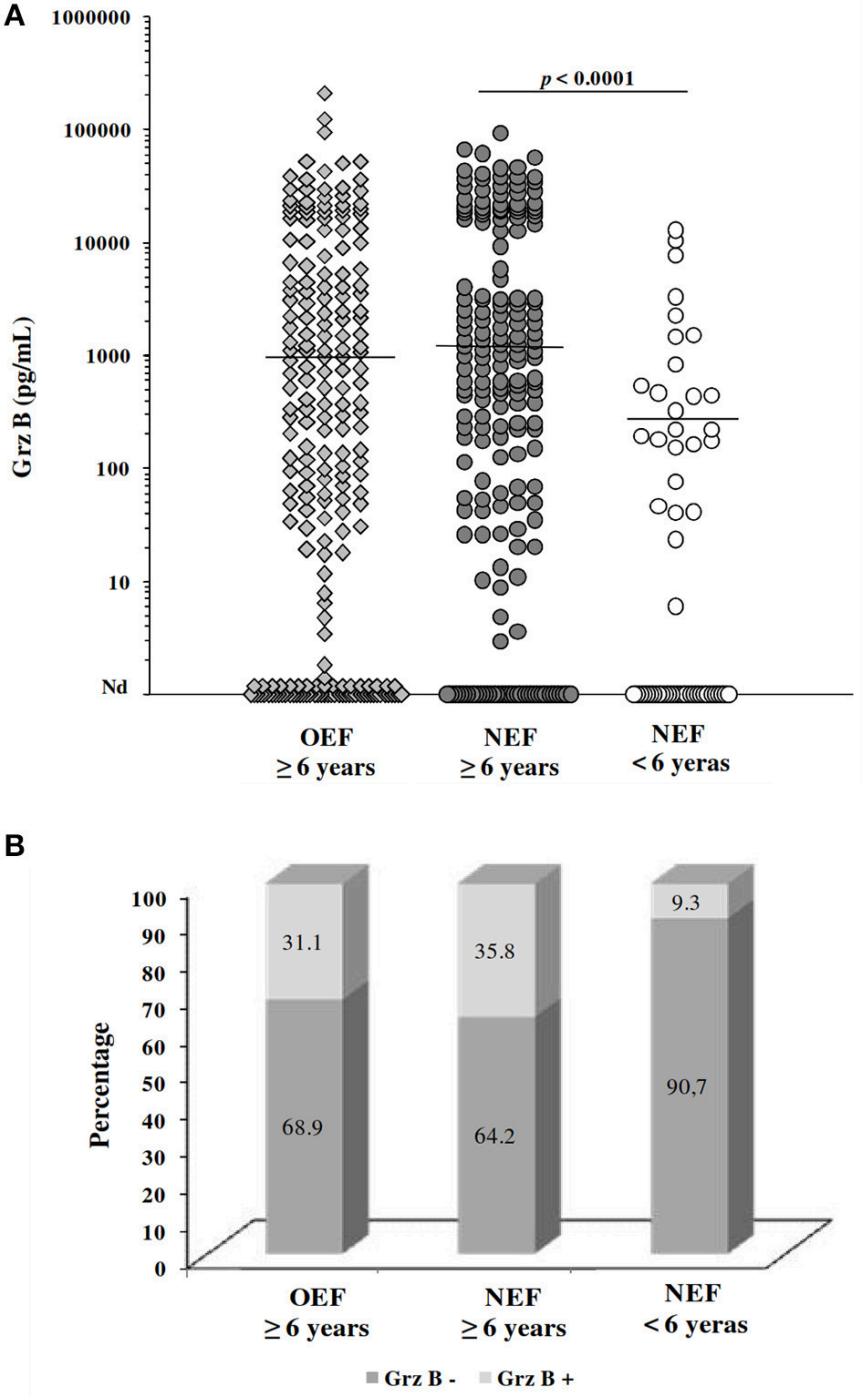
Evaluation of granzyme B levels for subjects included in the prospective study. **(A)** Grz B levels measured in culture supernatants of PBMCs from individuals, categorized basing on their home focus and their age. Horizontal bars represent median values. **(B)** Percentage of subjects showing high levels of GrzB (≥2,000 pg/mL; Grz B^+^) and those with levels <2,000 pg/mL (Grz B^−^). OEF: old endemic focus; NEF, New endemic focus.

Globally, Grz B was detected within 72.18% (327/453) of culture supernatants at variable levels ranging from 1.43 to 211 560.4 pg/mL (mean ± SD; 9158.5 ± 19246.6 pg/mL). Production of Grz B by PBMCs stimulated with *Leishmania* parasite was not associated with the age or the gender of subjects. Indeed, no correlation was found between subject's age and the Grz B levels (spearman rank correlation *r* = 0.175; *p* = 0.002). Likewise, the difference between Grz B levels measured within PBMCs from male subjects and those from female ones was not statistically significant (*p* = 0.449). Furthermore, no significant difference was found between Grz B levels detected within individuals from El Gtar (OEF; mean ± SD; 6,946 ± 20,295 pg/mL) and those within subjects from Souk Ejjdid (NEF; mean ± SD; 6,269 ± 13,181 pg/mL; *p* > 0.05). However, in the new endemic focus, Grz B levels were significantly higher within individuals over 6 years old comparing to those within children <6 years old (*p* < 0.0001; Figure 3A). Moreover, according to the cut-off of positivity of the Grz B levels, the percentage of individuals with high levels of Grz B (35. 8%) was greater in the group over 6 years old than in this under 6 years old (9.3%; Figure 3B). This could reflect the importance of frequency of the contact with the parasite (on time and intensity) on the development of the host's immune response.

### Association between clinical and immunological markers during ZCL

**Figure 4:**
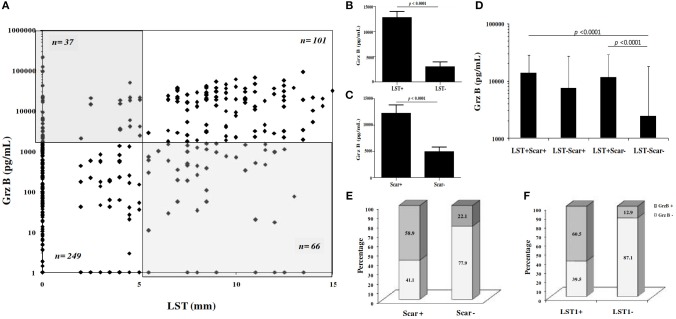
Association between granzyme B levels, LST reactivity and presence of typical ZCL scar. **(A)** Grz B level expressed in function of result of LST. *n* number of individual for each group. **(B–D)** Histograms represent mean values of Grz B levels measured in culture supernatants of PBMCs from individuals, categorized basing on categorical results of LST (LST^+^/LST^−^) and presence or absence of ZCL scars (scar^+^/scar^−^). **(E,F)** Categorial result for Grz B expression within individuals categorized basing on LST results (LST^+^/LST^−^) or presence or absence of scar (scar^+^/scar^−^). Grz B^+^: Grz B levels ≥2 ng/ml, Grz B^−^: Grz B levels <2 ng/ml. LST^+^, Diameter of induration ≥5 mm; LST^−^, Diameter of induration <5 mm.

We investigated association between Grz B levels, results of LST and the presence of typical ZCL scars. Statistical analysis showed a positive correlation between Grz B levels and the delayed hypersensitivity test (Spearman's rho correlation *r* = 0.523, *p* < 0.0001; Figure 4A). However, some discrepancy was noted between these tests. Indeed, we noted that 37 individuals showed variable levels of Grz B despite a negative LST (induration diameter < 5 mm) and conversely 66 individuals showed Grz B levels <2,000 pg/ml although they exhibited positive LST reactivity. As expected, levels of Grz B produced by PBMCs from individuals with a positive LST were higher comparing to those from individuals with negative LST (*p* < 0.0001; Figure 4B). Similarly, a significant difference was found between Grz B levels within individuals with ZCL scars (Scar^+^) and those without scar (Scar^−^; *p* < 0.0001; Figure 4C). Moreover, significant differences were found between Grz B levels measured in individuals with a negative LST and no ZCL scar (LST^−^Scar^−^) and those with a positive LST with scars (LST^+^ Scar^+^) or without scars (LST^+^ Scar^−^; *p* < 0.0001; Figure 4D).

In addition, we found that 138 individuals among the 453 individuals tested showed Grz B levels >2,000 pg /ml. These high levels were specifically found within individuals with positive LST and those with ZCL scars (Figures 4E,4F). As described in Table 3, statistical analysis showed a positive association between levels of Grz B and the positivity of LST (χ^2^ = 112.49, df = 1, *p* < 0.0001) and the presence of scars (χ^2^ = 49.26, df = 1, *p* < 0.0001).

**Table 3:** Odds ratios and confidence intervals describing the associations between putative markers of previous ZCL and the immune status at baseline for individuals of the study.

**1st test**	**Cut-off 1st test**	**2nd test**	**Cut-off 2nd Test**	***N***	**Odds ratio**	**Confidence intervals**	***p-*value**
LST	5	Grz B	2000	453	4.6^*^	3.3–6.4	<0.001
Scar	–	Grz B	2000	453	2.6^*^	2.03–3.3	<0.001
Scar	–	LST	5	453	2.7^*^	2.18–3.34	<0.001

LST, Leishmanin skin test; Cic, Presence/ansence of ZCL scar; Grz B, levels of granzyme measured within supernatant of PBMCs stimulated with L.major promastigotes; N, number of subject included in the study;

* *Statistically significant (p < 0.05)*.

### Incidence of ZCL among subjects of the prospective study after one transmission season

**Figure 5:**
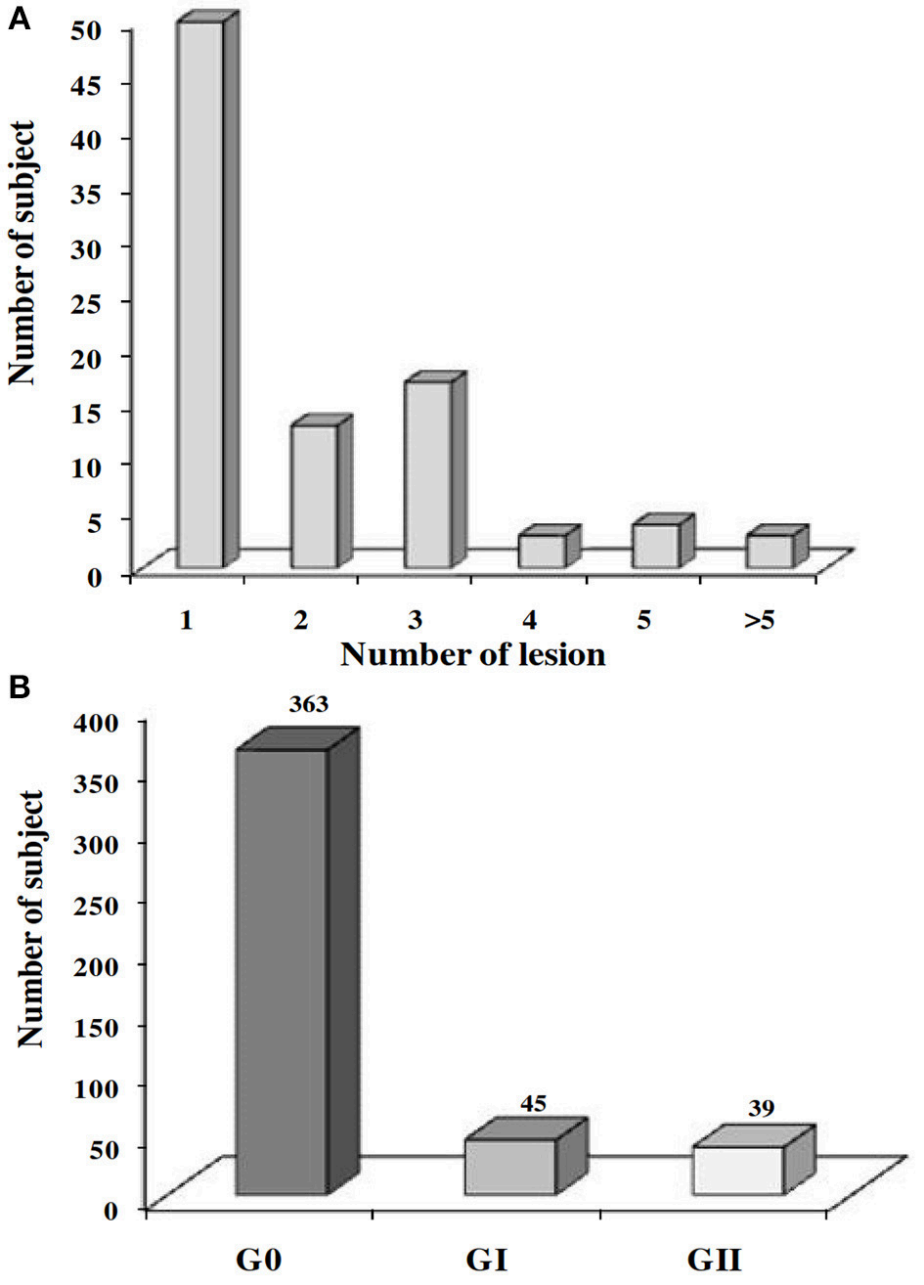
Prevalence of ZCL after one transmission season of *L. major*. Prevalence of the ZCL according to **(A)** the number of lesions developed or **(B)** the severity of the disease. G0, no ZCL lesion; GI, individuals developing a mild ZCL (severity score contained with the interval [0–20]); GII, individuals developing an intermediate or severe leishmaniasis (severity score ≥20).

To investigate the protective effect of the host's immune status on the development of ZCL, we performed a clinical follow-up of the 453 individuals, between the month of September 2001 and April 2002, to diagnose new lesions of ZCL. We noticed that, after one season of transmission, 90 among the 453 subjects (19.9%) developed new ZCL lesions. Number of lesions ranged from 1 to 9 (Figure 5A) and were mostly localized within limbs or face, with a total area ranging from 12.5 to 5985.6 mm^2^ (mean ± SD = 492.7 ± 784.8 mm^2^) and a mean duration of progression ranging from 71 to 439 days (mean ± SD = 143.7 ± 62 days).

To refine the analysis, we defined a new variable for the expression of the severity of the disease calculated basing on the total number of lesions, the total surface of lesions (taken at their maximum during the various visits) as well as the average duration of evolution of the lesions. The severity score is determined by the formula:

$$\begin{array}{l} \left[Average\;duration\;of\;lesions\;{\left(days\right)}^{*}Total\;lesion^{\prime }s\;area\;\left(mm^{2}\right)\right]\\ \;/\left[Number\;of\;lesions^{*}1000\right] \end{array}$$

We thus categorized ZCL patients into two groups: (GI) with a lesion's severity score comprised within the interval [0–20] and a second group (GII) with a severity score >20. Among the 90 individuals who developed a new ZCL lesion and for whom information on the spontaneous evolution and size of lesions were available (*n* = 84), 45 developed a mild form of the disease (GI) while 39 individuals presented intermediate or severe forms of the disease (GII; Figure 5B).

### Impact of the immune response in protection against infection by *L. major*

Finally, we evaluated the efficacy of the host's factors in protecting against *L. major* infection. These factors include the presence of typical ZCL scar, the positive LST reactivity and the induction of high levels of Grz B (≥2000 pg/ml). These factors showed a weak efficacy of protection against the development of ZCL lesion. Indeed, protective efficacy was 29, 15, and 22% for the presence of scar, positive LST reactivity and high Grz B levels with a non-significant *p* (Table 4).

**Table 4:** The relative risk (*RR*) of the different tests.

	**Development of ZCL**					**Severity of the disease**					
	**Cut-off**	***N***	**+/−^a^**	**+ZCL/−ZCL^b^**	***RR^c^***	***p***	**N**	**+/−^a^**	**Sv/–ZCL ^d^**	***RR^c^***	***p***
LST	5mm	453	167/286	90/363	0.85	0.466	402	144/258	39/363	0.392^*^	0.009^*^
Scar	–	453	105/348	90/363	0.713	0.209	402	93/309	39/363	0.38^*^	0.047^*^
Grz B	2 ng/mL	453	138/315	90/363	0.78	0.306	402	119/283	39/363	0.272^*^	0.003^*^

a *Number of subject positive for the test/Number of individuals negative for the test*.

b *Number of subject who developed ZCL lesions/Number of subjects without any ZCL lesion*.

c *RR, Relative Risk, RR < 1 indicates a decrease in the risk of ZCL and vice versa*.

d *Number of subject who developed an intermediate or severe ZCL (Sv)/Number of subjects without any ZCL lesion*.

* Statistically significant (p < 0.05)

*N, Number of tested subjects; LST, Leishmanin Skin Test; Scar +/−, presence/absence of ZCL scar; GrzB +/−, Grz B level ≥2 ng/ml or <2 ng/ml*.

*+/−Corresponding to the number of subject positive for the test/Number of subject negative for the test*.

**Figure 6:**
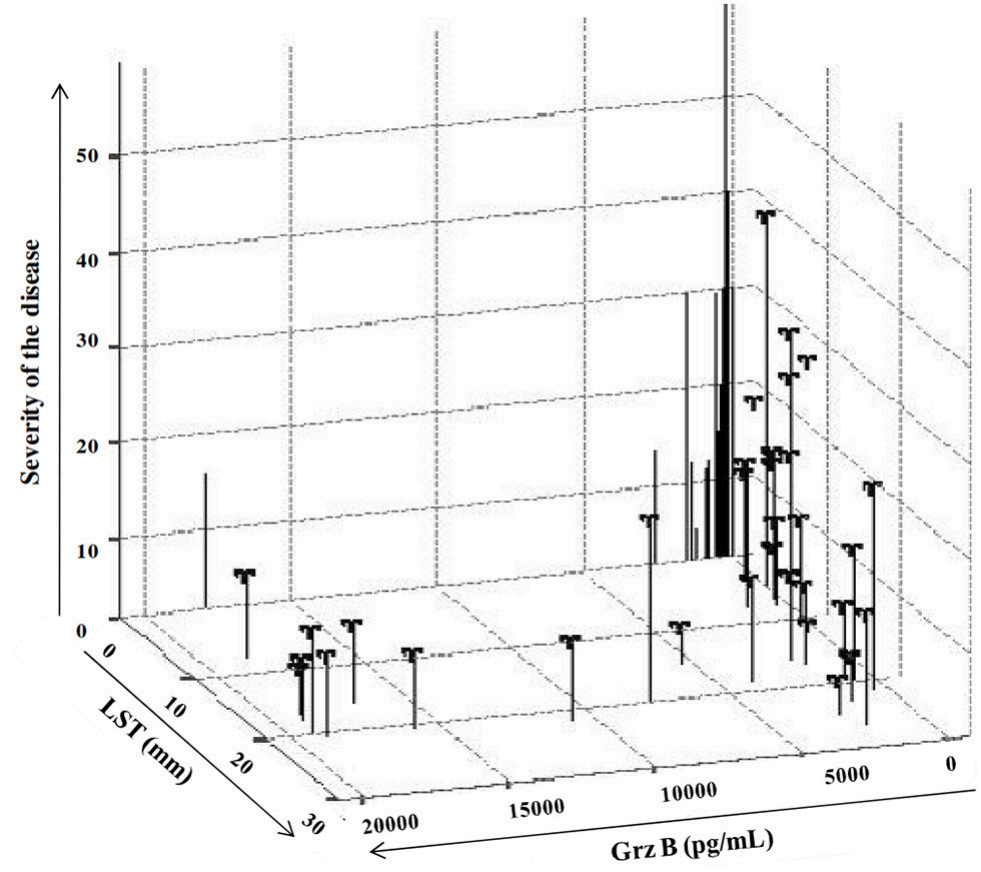
Impact of the host's immune response on the prevalence of ZCL. Expression of severity score in function of result of LST reactivity, expressed as diameter of induration (mm) and those of Grz B induction expressed in pg/mL.

When ZCL patients were categorized into two groups (GI and GII) according to the lesion's severity score, we showed that none of these parameters was protective against the development of mild ZCL (*RR* > 1). However, all these parameters were highly protective against the development of intermediate or severe leishmaniasis (Table 4). Accordingly, the incidence of the latter forms of leishmaniasis was significantly lower in individuals with typical ZCL scars (relative risk *RR* = 0.38, Fisher's exact *p* = 0.047) and presence of scar showed a protective efficacy of 62% (Table 4). Similarly, positive LST reactivity seems to be efficient in protecting against the development of intermediate or severe disease with a protective efficacy equal to 60% (*RR* = 0.39, Fisher's exact *p* = 0.009; Table 4). Strikingly, only 2.9% (4/137) of individuals with Grz B levels >2,000 pg/ml developed intermediate or severe forms of ZCL (Severity > 20) while 13.1% (18/137) developed a mild form of the disease with severity score comprised within the interval [0–20]. Thus high levels of Grz B showed an efficacy of 72.8% (relative risk *RR* = 0.27, Fisher's exact *p* = 0.003) to protect against such forms of leishmaniasis. Interestingly, together, the positivity of the LST and level of Grz B >2,000 pg/ml, leads to a better efficacy of protection against development of intermediate or severe leishmaniasis (efficacy is 83.6%). Indeed, only 2% (2/100) of LST^+^ individuals, with Grz B levels >2,000 pg/ml, developed intermediate or severe ZCL (Figure 6).

### Discussion

Conventionally, protection during leishmaniasis is associated with the development of a Th1-type immune response that can be demonstrated in humans by positive LST reactivity and specific *in vitro* T cell responses associated with IFN-γ production (Liew and O'Donnell, 1993; Sassi et al., 1999; Reithinger et al., 2007). We and others have demonstrated the involvement of cytotoxic T cell (CTL) response as part of the acquired adaptive immune response developed against the parasite *Leishmania* (da Conceição-Silva et al., 1994; Brodskyn et al., 1997; Marry et al., 1999; Russo et al., 1999; Bousoffara et al., 2004). However, the role of the CTL response during *Leishmania* infections is still controversial. In humans, clinical studies show the presence of a large number of CD8^+^ T cells in the lesions as well as in blood of ZCL patients during the acute phase, but also during the healing process (Da-Cruz et al., 1994, 2002, 2005; Gaafar et al., 1999; Bottrel et al., 2001). In addition, *L. major* infection induces Th1 and CD8^+^ T cells in human patients and both responses are associated with disease resolution (Nateghi Rostami et al., 2010). Nevertheless, other studies have shown that cytotoxicity is one of the main mechanisms underlying disease induced by *L. braziliensis* infection (Faria et al., 2009; Dantas et al., 2013; Novais et al., 2013; Santos et al., 2013; Cardoso et al., 2015; Ferraz et al., 2015, 2017; Novais and Scott, 2015). Herein, we used the release of Grz B by PBMC in response to live *L.major* promastigotes as marker of cytotoxicity and clearly demonstrated that Grz B is a good marker of immunity to *L.major* infection, but, most importantly it constitutes a good correlate for protection against intermediate and severe form of CL due to *L. major* infection. This result is extremely important for vaccine development and evaluation.

In this study, we first showed that in endemic foci of *L. major* infection subjects having a previous contact with the parasite showed the presence of *Leishmania*-specific cytotoxicity. Indeed, compared to healthy individuals living outside endemic area for *L. major* transmission, those living in these areas, including subjects with active or healed ZCL but also those apparently healthy with no ZCL scars and no LST reactivity (Scar^−^ LST^−^) showed high levels of Grz B. Interestingly, even in the absence of LST reactivity, Grz B induction may constitute an indicator of a previous contact with the parasite and may reflect an eventual role in control of *Leishmania* infection. This data is contradictory with those described by Cordosa and collaborators showing the involvement of CD8^+^T cells producing IFN-g rather than those with cytotoxic activity in the control of *Leishmania* infection (Cardoso et al., 2015). One explanation could be a difference in the nature of Grz B-producing cells between subjects with history of ZCL and those with subclinical infection. Accordingly, we have previously shown that in healed ZCL subjects (Scar^+^LST^+^), the main source of GrB produced in response to stimulation with *Leishmania*-antigens is CD4^+^ T cells, including T regulatory cells. These CD4^+^ T cells were different from the Th1 cells producing IFN-γ (Naouar et al., 2014).

Furthermore, the higher peripheral Grz B levels in individuals healed from ZCL compared to those with active disease might be attributed to the homing of Grz B-producing cells to the site of infection in the latter group. Accordingly, we have demonstrated high levels of Grz B mRNA as well as the presence of double positive CD8^+^ Grz B^+^ T cells within active ZCL lesions (Boussoffara et al., submitted). In American cutaneous leishmaniasis (ACL), the percentage of CD8^+^ T cells were higher in lesions compared to blood (Conceição-Silva et al., 1990; Da-Cruz et al., 2005). This, associated with an increase in *Leishmania*-specific T cells in lesions, have been attributed to the migratory process between these compartments Moreover, a predominance of CD4^+^ and CD8^+^ effector memory T cells (T_EM_-CD45RO^+^CCR7^−^) have been shown in ACL lesions. An enrichment of T_EM_ for both CD4^+^ and CD8^+^ T cells sets were observed comparing to blood (de Oliveira Mendes-Aguiar et al., 2016).

In a next step, the measurement of Grz B levels in culture supernatants of PBMCs stimulated with *L. major* promastigotes was performed during the prospective study of a cohort of 453 individuals living in *L.major* transmission area. We, thus, recovered in the beginning of the study (before the season of *L. major* transmission) the associations between the different parameters indicators of an earlier contact with the *Leishmania* parasite (presence of typical ZCL scars, LST reactivity and production of Grz B). Our results showed some discrepancies between production of Grz B and scars or LST reactivity. The discrepancy between production of Grz B and the presence or absence of scars might be explained by the fact that lesions of ZCL could disappear without leaving scars and that scars of other pathologies could be taken for ZCL scars. However, the discrepancy between Grz B and LST reactivity seems to be more relevant. Thus, a large fraction of individuals (*n* = 134) living in the endemic area of leishmaniasis showed a diameter of induration equals to zero but variable levels of Grz B which are sometimes very high. This is consistent of the striking results found during the validation of the Grz B test. Indeed, we were struck by the differences in Grz B levels between the so-called “naïve” (Scar^−^ LST^−^) living in an endemic area of leishmaniasis vs. those living outside the endemic areas. These results are extremely important for the selection of naive populations in endemic areas, especially when including adults in vaccination trials. In fact, the selection of individuals Scar^−^LST^−^ does not exclude an eventual previous contact with the parasite which could be tracked otherwise by the Grz B analysis.

Secondly, we explored the protective role of the cytotoxic effector against the development of the disease. Since ZCL is heterogeneous in terms of disease severity (number and size of lesions, duration of spontaneous healing), we hypothesized that host immune response will not protect against the development of leishmaniasis but rather against the severe forms of it. Our objective was achieved through the monitoring of the cohort included in our prospective study. We analyzed the predictive value of a cytotoxic response (investigated by the Grz B assay) in resistance to *L. major* infection, as well as the positive LST reactivity, usually used as a protective correlate. Immune response was evaluated at the baseline (before the parasite transmission season) and the development of new ZCL cases was monitored actively during the season of emergence of the disease. A gradation of the disease was also made by calculating a new parameter, severity of the disease, basing on the size of lesions as well as their total duration of evolution.

During the season of emergence of the disease, we diagnosed a total of 90 new cases of ZCL among the 453 individuals tested, with mild, intermediate or high severity score. Interestingly, we noticed a significant number (*n* = 45) of individuals that develop a simple ZCL characterized by a unique or low number of lesion(s) and a rapid spontaneous healing (severity score <20). Surprisingly, we found that neither the positivity of the LST, nor the presence of ZCL scar, nor the cytotoxic activity (Grz B level ≥2,000 pg/ml), were protective against the development of the disease. This can be explained by the fact that in our study, patients have been actively monitored allowing the diagnosis of mild forms of ZCL, often ignored by the patient himself. However, the positive LST reactivity, the presence of ZCL scar and also the cytotoxic activity were protective against the development of intermediate or severe forms of ZCL. Accordingly, we showed that a Grz B level >2,000 pg/ml has a protective efficacy of 72.8% against the development of intermediate or severe forms of ZCL. Such efficacy was greater than those obtained with the presence of ZCL scar and the positivity of LST (62 and 60%, respectively). Interestingly, together positivity of the LST and a cytotoxic response (Grz B level ≥2,000 pg/ml) confer an excellent protection (>80% efficiency) against intermediate or severe forms of ZCL.

Altogether, our study clearly demonstrated that Grz B production may be an indicator of a previous contact with *L.major* although the negativity of LST and the absence of scars. Such parameter could be used in vaccination trials for the selection of naive populations living in endemic areas. Moreover, our data show that the preexistence of a parasite-specific cytotoxic immune response may confer protection against the development of intermediate or severe forms of ZCL. These results are of crucial importance for anti-*Leishmania* vaccine design and more generally for the evaluation of their protective efficacy.

## Author contributions

TB, KD, HL, and AB conceived and designed the experiments. TB performed the experiments. MM carried out the clinical monitoring. TB and SC performed the statistical analyses. TB, MB, and HL wrote the manuscript with the input and approval of all other coauthors.

### Conflict of interest statement

The authors declare that the research was conducted in the absence of any commercial or financial relationships that could be construed as a potential conflict of interest.

## References

[B1] Alimohammadian M. H. Khamesipour A. Darabi H. Firooz A. Malekzadeh S. Bahonar A. (2002). The role of BCG in human immune responses induced by multiple injections of autoclaved *Leishmania major* as a candidate vaccine against leishmaniasis. Vaccine 2, 174–180. 10.1016/S0264-410X(02)00458-9 12450691

[B2] Armijos R. X. Weigel M. M. Calvopina M. Hidalgo A. Cevallos W. Correa J. (2004). Safety, immunogenicity, and efficacy of an autoclaved *Leishmania amazonensis* vaccine plus BCG adjuvant against New World cutaneous leishmaniasis. Vaccine 22, 1320–1326. 10.1016/j.vaccine.2003.06.002 15003662

[B3] Belkaid Y. Von Stebut E. Mendez S. Lira R. Caler E. Bertholet S.. (2002). CD8^+^T cells required for primary immunity in C57BL/6 mice following low dose, intradermal challenge with *Leishmanai major*. J. Immunol. 168, 3992–4000. 10.4049/jimmunol.168.8.3992 11937556

[B4] Ben Ismail R. Ben Rachid M. (1989). Epidemiology of leishmaniasis in Tunisia, in Tropical Communicable Diseases, ed AUPELF-UREE (Paris: John Libbey Eurotext), 73–80.

[B5] Ben Salah A. Louzir H. Chlif S. Mokni M. Zaâtour A. Raouène M.. (2005). The predictive validity of naturally acquired delayed-type hypersensitivity to leishmanin in resistance to *Leishmania major*–associated cutaneous leishmaniasis. J. Inf. Dis. 192, 1981–1987. 10.1086/498042 16267771

[B6] Bettaieb J. Nouira M. (2017). Epidemiology of cutaneous leishmaniasis in Tunisia, in The Epidemiology and Ecology of Leishmaniasis, ed Claborn D. (InTechOpen), 79–90. 10.5772/65788

[B7] Bettaieb J. Toumi A. Chlif S. Chelghaf B. Boukthir A. Gharbi A.. (2014). Prevalence and determinants of *Leishmania major* infection in emerging and old foci in Tunisia. Parasit. Vectors 7:386. 10.1186/1756-3305-7-386 25142220 PMC4262385

[B8] Bottrel R. L. Dutra W. O. Martins F. A. Gontijo B. Carvalho E. Barral-Netto M.. (2001). Flow cytometric determination of cellular sources and frequencies of key cytokine-producing lymphocytes directed against recombinant LACK and soluble *Leishmania* antigen in human cutaneous leishmaniasis. Infect. Immun. 69, 3232–3239. 10.1128/IAI.69.5.3232-3239.2001 11292745 PMC98281

[B9] Bousoffara T. Louzir H. Ben Salah A. Dellagi K. (2004). Analysis of granzyme B activity as a surrogate marker of *Leishmania*-specific cell mediated cytotoxicity in zoonotic cutaneous leismaniasis. J. Infect. Dis 189:126571263. 10.1086/382031 15031796

[B10] Brodskyn C. I. Barral A. Boaventura V. Carvalho E. Barrel-Netto M. (1997). Parasite-driven *in vitro* human lymphocyte cytotoxicity against autologous infected macrophages from mucosal leishmaniasis. J. Immunol. 159, 4467–4473. 10.1111/j.1365-3083.2007.01964.x 9379046

[B11] Cardoso T. M. Machado A. Costa D. L. Carvalho L. P. Queiroz A. Machado P.. (2015). Protective and pathological functions of CD8^+^ T Cells in *Leishmania braziliensis* infection. Infect. Immun. 83, 898–906. 10.1128/IAI.02404-14 25534940 PMC4333467

[B12] Conceição-Silva F. Dórea R. C. Pirmez C. Schubach A. Coutinho S.G. (1990). Quantitative study of Leishmania braziliensis reactive T cells in peripheral blood and in the lesions of patients with American mucocutaneous leishmaniasis. Clin. Exp. Immunol. 79, 221–226. 10.1111/j.1365-2249.1990.tb05182.x 2311299 PMC1534771

[B13] da Conceição-Silva F. Perlaza B. Louis J. A. Romero P. (1994). *Leishmania major* infection in mice primes for specific major histocompatibitlity complex class I–restricted cytotoxic T cell responses. Eur. J. Immunol. 24, 2813–2817. 10.1002/eji.1830241135 7957573

[B14] Da-Cruz A. M. Bertho A. L. OliveiraNeto M. P. Coutinho S. G. (2005). Flow cytometric analysis of cellular infiltrate from American tegumentary leishmaniasis lesions. Br. J. Dermatol. 153, 537–543. 10.1111/j.1365-2133.2005.06647.x 16120139

[B15] Da-Cruz A. M. Bittar R. Mattos M. Oliveira-Neto M. P. Nogueira R. Pinho-Ribeiro V.. (2002). T-cell-mediated immune responses in patients with cutaneous or mucosal leishmaniasis: long-term evaluation after therapy. Clin. Diagn. Lab. Immunol. 9, 251–256. 10.1128/CDLI.9.2.251-256.2002 11874860 PMC119941

[B16] Da-Cruz A. M. Conceicao-Silva F. Bertho A. L. Coutinho S. G. (1994). *Leishmania* reactive CD4^+^ and CD8^+^ T cells associated with cure of human cutaneous leishmaniasis. Infect. Immun. 62, 2614–2618. 7910596 10.1128/iai.62.6.2614-2618.1994 PMC186553

[B17] Dantas M. L. Oliveira J. C. Carvalho L. Passos S. T. Queiroz A. Machado P.. (2013). CD8^+^ T cells *in situ* in different clinical forms of human cutaneous leishmaniasis. Rev. Soc. Bras. Med. Trop. 46, 728–734. 10.1590/0037-8682-0174-2013 24474014 PMC4105155

[B18] Davies C. R. Lianos-Cuentas E. A. Pyke S. D. M. Dye C. (1995). Cutaneous leishmaniasis in the Peruvian Andes: an epidemiological study of infection and immunity. Epidemiol. Infect. 114, 297–318. 10.1017/S0950268800057964 7705493 PMC2271273

[B19] de Luca P. M. Mayrink W. Pinto J. A. Coutinho S. G. Santiago M. A. Toledo V. P.. (2001). A randomized double-blind placebo-controlled trial to evaluate the immunogenicity of a candidate vaccine against American tegumentary leishmaniasis. Acta. Trop. 80, 251–260. 10.1016/S0001-706X(01)00181-4 11700183

[B20] de Oliveira Mendes-Aguiar C. Vieira-Gonçalves R. Guimarães L. H. de Oliveira-Neto M. P. Carvalho E. M. Da-Cruz A. M. (2016). Effector memory CD4^+^ T cells differentially express activation associated molecules depending on the duration of American cutaneous leishmaniasis lesions. Clin. Exp. Immunol. 185, 202–209. 10.1111/cei.12798 27059407 PMC4955010

[B21] Duthie M. S. Raman V. S. Piazza F. M. Reed S. G. (2012). The development and clinical evaluation of second-generation leishmaniasis vaccines. Vaccine 30, 134–141. 10.1016/j.vaccine.2011.11.005 22085553 PMC3359766

[B22] Faria D. R. Souza P. E. Duraes F. V. Carvalho E. M. Gollob K. J. Machado. (2009). Recruitment of CD8^+^ T cells expressing granzyme A is associated with lesion progression in human cutaneous leishmaniasis. Parasit. Immunol. 31, 432–439. 10.1111/j.1365-3024.2009.01125.x 19646207 PMC2764276

[B23] Ferraz R. Cunha C. F. Gomes-Silva A. Schubach A. O. Pimentel M. I. Lyra M. R. (2015). Apoptosis and frequency of total and effector CD8^+^ T lymphocytes from cutaneous leishmaniasis patients during antimonial therapy. BMC Infect. Dis. 19:74 10.1186/s12879-015-0799-x PMC4338827 25870976

[B24] Ferraz R. Cunha C. F. Pimentel M. I. F. Ferraz R. Lyra M. R. Pereira-Da-Silva T.. (2017). CD3^+^CD4^neg^CD8^neg^ (double negative) T lymphocytes and NKT cells as the main cytotoxic-related-CD107a^+^cells in lesions of cutaneous leishmaniasis caused by *Leishmania (Viannia) braziliensis*. Parasit. Vectors 10:219. 10.1186/s13071-017-2152-2 28468680 PMC5415843

[B25] Gaafar A. Veress B. Permin H. Kharazmi A. Theander T. G. El Hassan A. M. (1999). Characterization of the local and systemic immune responses in patients with cutaneous leishmaniasis due to *Leishmania major*. Clin. Immunol. 91, 314–320. 10.1006/clim.1999.4705 10370377

[B26] Guirges S. Y. (1971). Natural and experimental re-infection of man with oriental score. Ann Trop Med Parasitol. 65, 197–205. 4253634 10.1080/00034983.1971.11686746

[B27] Kébaïer C. Louzir H. Chenik M. Ben Salah A. Dellagi K. (2001). Heterogeneity of wild *Leishmania major* isolates in experimental murine pathogenicity and specific immune response. Infect. Immun. 69, 4906–4915. 10.1128/IAI.69.8.4906-4915.2001 11447167 PMC98581

[B28] Khalil E. A. El Hassan A. M. Zijlstra E. E. Mukhtar M. M. Ghalib H. W. Musa B.. (2000). Autoclaved *Leishmania major* vaccine for prevention of visceral leishmaniasis: a randomised, double-blind, BCG-controlled trial in Sudan. Lancet 356, 1565–1569. 10.1016/S0140-6736(00)03128-7 11075771

[B29] Liew F. Y. O'Donnell C. A. (1993). Immunology of leishmaniasis. Adv. Parasitol. 32, 161–259. 8237615 10.1016/s0065-308x(08)60208-0

[B30] Marry C. Auriault V. Faugere B. Dessein A. J. (1999). Control of *Leishmania infantum* infection is associated with CD8^+^ and gamma interferon- and interleukin-5-producing CD4^+^ antigen-specific T cells. Infect. Immun. 67, 5559–5566. 10531200 10.1128/iai.67.11.5559-5566.1999 PMC96926

[B31] Momeni Boroujeni A. Aminjavaheri M. Moshtaghian B. Momeni A. Momeni A. Z. (2013). Reevaluating leishmanin skin test as a marker for immunity against cutaneous leishmaniasis. Int. J. Dermatol. 52, 827–830. 10.1111/j.1365-4632.2012.05850.x 23621513

[B32] Naouar I. Boussoffara T. Ahmed M. B. Hmida N. B. Gharbi A. Gritli S.. (2014). Involvement of different CD4^+^ T cell subsets producing granzyme B in the immune response to *Leishmania major* antigens. Mediators. Inflamm. 2014:636039. 10.1155/2014/636039 25104882 PMC4102068

[B33] Nateghi Rostami M. Keshavarz H. Edalat R. Sarrafnejad A. Shahrestani T. Mahboudi F.. (2010). CD8^+^ T cells as a source of IFN-gamma production in human cutaneous leishmaniasis. PLoS. Negl. Trop. Dis. 4:e845. 10.1371/journal.pntd.0000845 20967288 PMC2953482

[B34] Novais F. O. Carvalho L. P. Graff J. W. Beiting D. P. Ruthel G. Roos D. S.. (2013). Cytotoxic T cells mediate pathology and metastasis in cutaneous leishmaniasis. PLoS Pathog. 9:e1003504. 10.1371/journal.ppat.1003504 23874205 PMC3715507

[B35] Novais F. O. Scott P. (2015). CD8^+^ T cells in cutaneous leishmaniasis: the good, the bad, and the ugly. Semin Immunopathol. 37, 251–259. 10.1007/s00281-015-0475-7 25800274 PMC4439344

[B36] Reithinger R. Dujardin J. C. Louzir H. Pirmez C. Alexander B. Brooker S. (2007). Cutaneous leishmaniasis. Lancet Infect. Dis. 7, 581–596. 10.1016/S1473-3099(07)70209-8 17714672

[B37] Rhee E. G. Mendez S. Shah J. A. Wu C. Y. Kirman J. R. Turon T. N.. (2002). Vaccination with heat-killed *Leishmania* antigen or recombinant leishmanial protein and CpG oligodeoxynucleotides induces long-term memory CD4^+^ and CD8^+^ T cell responses and protection against *Leishmania major* infection. J. Exp. Med. 195, 1565–1573. 10.1084/jem.20020147 12070284 PMC2193566

[B38] Rothman K. J. Greenland S. Lash T. L. (2008). Measure of effect and measures of associations, in Modern Epidemiology, eds Williams and Wilkins L. (Philadelphia, PA), 51–70.

[B39] Ruiz J. H. Becker I. (2007). CD8 cytotoxic T cells in cutaneous leishmaniasis. Parasit. Immunol. 29, 671–678. 10.1111/j.1365-3024.2007.00991.x 18042173

[B40] Russo D. M. Chakrabarti P. Higgins A. Y. (1999). *Leishmania:* naïve human T cells sensitized with promastigote antigen and IL-12 develop into potent Th1 and CD8^+^ cytotoxic effectors. Exp Parasitol. 93, 161–170. 10.1006/expr.1999.4452 10529358

[B41] Santos C. S. Boaventura V. Ribeiro Cardoso C. Tavares N. Lordelo M. J. (2013). CD8^+^ granzyme B^+^-mediated tissue injury vs. CD4^+^IFN gamma^+^-mediated parasite killing in human cutaneous leishmaniasis. J. Invest. Dermatol. 133, 1533–1540. 10.1038/jid.2013.4 23321919 PMC3667352

[B42] Sassi A. Louzir H. Ben Salah A. Mokni M. Ben Osman A. Dellagi K. (1999). Leishmanin skin test lymphoproliferative response and cytokine production after symptomatic or asymptomatic *Leishmania major* infection in Tunisia. Clin. Exp. Immunol. 116, 127–132. 10209516 10.1046/j.1365-2249.1999.00844.x PMC1905205

[B43] Sharifi I. Fekri A. R. Aflatonian M. R. Khamesipour A. Nadim A. Mousavi M. R. (1998). Randomised vaccine trial of single dose of killed *Leishmania major* plus BCG against anthroponotic cutaneous leishmaniasis in Bam, Iran. Lancet 35, 1540–1543. 10.1016/S0140-6736(98)09552-X 10326536

[B44] Spaeny-Dekking E. H. A. Hanna W. L. Wolbink A. M. Wever P. C. Kummer A. J. Swaak A. J. G. (1998). Extracellular granzymes A and B in humans: direction of native species during CTL responses *in vitro* and *in vivo. J*. Immunol. 160, 3610–3616. 9531325

[B45] Stäger S. Rafati S. (2012). CD8^+^ T cells in *Leishmania* infections: friends or foes? Front. Immunol. 24:5. 10.3389/fimmu.2012.00005 PMC3342007 22566891

[B46] van Belle G. Fisher L. D. Heagerty P. J. Lumley T. S. (2004). Counting Data, in Biostatistics: A Methodology for the Health Sciences, (New York, NY: Wiley), 151–207.

